# Fractionation of carbohydrate and protein content of some forage feeds of ruminants for nutritive evaluation

**DOI:** 10.14202/vetworld.2015.197-202

**Published:** 2015-02-19

**Authors:** Lalatendu Keshary Das, S. S. Kundu, Dinesh Kumar, Chander Datt

**Affiliations:** 1 Veterinary Dispensary, Kalampur, Kalahandi, Odisha, India; 2 Division of Dairy Cattle Nutrition, National Dairy Research Institute, Karnal, Haryana, India; 3 Division of Animal Nutrition, Indian Veterinary Research Institute, Izatnagar, Uttar Pradesh, India

**Keywords:** carbohydrate and protein fractions, green fodders, range herbages, ruminants

## Abstract

**Aim:**

To evaluate some forage feeds of ruminants in terms of their carbohydrate (CHO) and protein fractions using Cornell Net Carbohydrate and Protein System (CNCPS).

**Materials and Methods:**

Eleven ruminant feeds (six green fodders - maize, oat, sorghum, bajra, cowpea, berseem and five range herbages - para grass, guinea grass, hedge lucerne, setaria grass and hybrid napier) were selected for this study. Each feed was chemically analyzed for proximate principles (dry matter, crude protein [CP], ether extract, organic matter and ash), fiber fractions (neutral detergent fiber, acid detergent fiber, acid detergent lignin, cellulose and hemicellulose), primary CHO fractions (CHO, non-structural CHO, structural CHO and starch) and primary protein fractions (neutral detergent insoluble CP, acid detergent insoluble CP, non-protein nitrogen and soluble protein). The results were fitted to the equations of CNCPS to arrive at various CHO (CA - fast degrading, CB_1_ - intermediate degrading, CB_2_ - slow degrading and CC - non-degrading or unavailable) and protein (PA - instantaneously degrading, PB_1_ - fast degrading, PB_2_ - intermediate degrading, PB_3_ - slow degrading and PC - non-degrading or unavailable) fractions of test feeds.

**Results:**

Among green fodders, cowpea and berseem had higher CA content while except hedge lucerne all range herbages had lower CA values. CB_1_ content of all feeds was low but similar. All feeds except cowpea, berseem, and hedge lucerne contained higher CB_2_ values. Oat among green fodders and hybrid napier among range herbages had lower CC fraction. Feeds such as bajra, cowpea, berseem and the setaria grass contained lower PA fraction. All green fodders had higher PB_1_ content except maize and cowpea while all range herbages had lower PB_1_ values except hedge lucerne. Para grass and hybrid napier contained exceptionally low PB_2_ fraction among all feeds. Low PC contents were reported in oat and berseem fodders.

**Conclusion:**

Based on our findings, it was concluded that feeds with similar CP and CHO content varied significantly with respect to their CHO and protein fractions. Due to lower CC fraction, oat and hybrid napier were superior feeds in terms of CHO supply to ruminants. Similarly, among all feeds oat and berseem had a lower PC fraction, thus were considered good sources of protein for ruminants.

## Introduction

Forages usually constitute the major portion of the ruminant feeds in our country. Due to acute shortage of concentrate feeds for animals, the livestock farming in India still relies heavily on forage feed resources [1]. Dry forages such as straw, stover, husk, etc. are nutritionally very poor and usually fulfill only the appetite of the animals. However, green forages such as fodders and range herbages are generally adequate in meeting the requirements of maintenance and moderate levels of production in ruminants. Thus, their proper nutritive evaluation is the need of the hour for their optimum utilization in low producing animals of our country. Dietary nutrients particularly carbohydrates (CHO) and proteins are often heavily modified in rumen before their presentation to the animal for real digestive processes. Knowledge of potential rumen degradability of feed fractions is key to assess their nutritive values and extent of utilization in ruminants. Conventional proximate and detergent analysis procedures do not meet these criteria. Hence, a system including above factors will be the most scientific way of feed analysis. The Cornell Net Carbohydrate and Protein System (CNCPS) as described by Fox *et al*. [2] seems to be the answer to the existing feed analytical limitations; because it accounts for the effects of variation due to feed CHO and protein fractions, their relative ruminal degradation rates and ultimately their rate of passage through the intestine. The system has been further modified to cater to the needs of present day new ruminant feeds [3,4]. Reports regarding fractions of various classes of Indian feeds are available. Certain forages were evaluated by research workers like Trivedi *et al*. [5], Kamble *et al*. [6] and Singh *et al*. [7]. But there exists huge variations among the published reports; therefore further information is needed to update the feed database of Indian origin.

Thus, the present study was undertaken to evaluate certain forage feeds of ruminants as per CNCPS model and to assess the acceptability of this model in the preparation of balanced rations for dairy animals.

## Materials and Methods

### Ethical approval

The experimental design and plan of the present study were duly approved by the academic council of National Dairy Research Institute (NDRI), Karnal, Haryana. As there was no direct involvement of animals in the experiment, no ethical permission was required.

### Collection and processing of green forage samples

Samples of green forages comprised of fodders and range herbages (maize - *Zea mays*, oat - *Avena sativa*, sorghum - *Sorghum bicolor*, bajra - *Pennisetum typhoides*, cowpea - *Vigna unguiculata*, berseem - *Trifolium alexandrinum*, para grass - *Brachiaria mutica*, guinea grass - *Panicum maximum*, hedge lucerne - *Desmanthus virgatus*, setaria grass - *Setaria pumila* and hybrid napier - *Pennisetum purpureum*) were collected from 2 locations: The experimental fodder farm of NDRI and Indian Grassland and Fodder research Institute. Samples (leaves and thin stem included) were thoroughly mixed and a representative amount of about 500 g of each feed was pooled. Samples were oven dried for 48 h at 60°C and then ground to pass through a 1 mm sieve in an electrically operated Willey mill. The ground samples were stored for further analyses.

### Chemical analyses

Samples of forage feeds were analyzed for dry matter (DM), crude protein (CP), ether extract (EE), organic matter (OM) and ash as per the standard procedures of AOAC [8]. Neutral detergent fiber (NDF) and acid detergent fiber (ADF) were determined by the procedures of Van Soest *et al*. [9]. Heat stable alpha amylase and sodium sulfite were not used in NDF determination. Both NDF and ADF were expressed exclusive of ash. Lignin was determined by solublization of cellulose with 72% (w/w) sulfuric acid in ADF residue. The difference between ADF and lignin in the sequential analysis was the cellulose content of test feeds. Difference between NDF and ADF was the indirect measure of hemicellulose (HC).

### CHO fractionation

Total CHO (%) content was determined by subtracting CP (%), EE (%) and ash (%) from 100. The non-structural CHO (NSC) content (%) was estimated directly from the following formula: 100 - [CP% + EE%+ {NDF% - neutral detergent insoluble CP (NDICP)%} + ash%]. Difference between total CHO and NSC was the indirect measure of structural CHO (SC) content of test feeds. Starch estimation in the feeds was done as per the procedure of Sastry *et al*. [10]. Samples were extracted with ethyl alcohol, solubilized with perchloric acid and then treated with anthrone-sulfuric acid to determine the starch content colorimetrically using standard glucose. ADL (% NDF) and starch (% NSC) contents were computed indirectly from their DM basis values. Equations of Sniffen *et al*. [11] were used to calculate CNCPS CHO fractions, which classifies CHO contents according to degradation rate into four fractions being CA (sugars and organic acids), CB_1_ (starch and pectins), CB_2_ (available cell wall content) and CC (unavailable lignin bound cell wall content).

### Protein fractionation

NDICP, acid detergent insoluble CP (ADICP), non-protein nitrogen (NPN) and soluble protein (SP) content of test feeds were estimated as per Licitra *et al*. [12]. ADICP fraction was assumed to be indigestible. CP of NPN origin was estimated as the difference between total CP and CP of true protein (TP) origin precipitated with 10% trichloroacetic acid solution. Similarly, SP content was calculated as the difference between total CP and buffer insoluble CP estimated with borate phosphate buffer (pH 6.7-6.8) and freshly prepared (1 g/10 ml) sodium azide solution. NPN (% SP) contents of feeds were computed indirectly from their DM basis values. Equations of Sniffen *et al*. [11] were used to calculate CNCPS protein fractions, which classifies protein contents according to degradation rate into five fractions being PA (NPN compounds), PB_1_ (globulins mainly), PB_2_ (albumins mainly), PB_3_ (prolamine, cell wall protein, denatured protein) and PC (Maillard protein, lignin and tannin bound protein).

### Statistical analyses

The results obtained were subjected to statistical analyses using software package SPSS version 16.0 [13]. Means were compared using one-way analysis of variance (ANOVA) test at 5% level of significance.

## Results and Discussion

### Chemical constituents

Chemical constituents of forage feeds (Table-1) revealed that the DM content of all feeds was in the range of 15-20% though higher DM content was observed in range herbages. OM and ash contents in all feeds were more or less similar. Feeds of leguminous origin such as cowpea, berseem, and hedge lucerne had higher CP values while others had lower but similar CP contents. Average EE content was slightly higher in green fodders than range herbages while average NDF, ADF and cellulose contents were significantly higher in range herbages. Feeds of leguminous origin such as cowpea, berseem, and hedge lucerne had lower NDF contents than other feeds, just reverse of the trend observed in CP values. Wadhwa *et al*. [14] reported slightly less CP and NDF content for maize and sorghum fodder than our findings. On the contrary, the CP value of bajra fodder was less, and the NDF value was more than that of the present study. However, the values were comparable. Islam *et al*. [15] reported significantly lower CP and higher NDF value in the oat fodder, which might possibly due to higher stage of maturity during the time of study. Agza *et al*. [16] evaluated seven cultivars of cowpea fodder and found a mean CP and NDF contents of 23.9 and 44.4%, respectively. Our findings were significantly different as the CP value was quite lower and the NDF value was higher, but comparable to that of above report. Mandal and Banerjee [17] evaluated nutritive value of berseem in sheep and arrived at a value of 14.5% for CP content of the berseem fodder, which was lower than our findings. However findings of Prusty *et al*. [18] were in conformation with present results though the NDF content was a bit higher. CP content in para and guinea grass as that reported by Raja Kishore and Parthasarathy [19] was higher than our observations while the NDF content in these grasses were reported less. Khanum *et al*. [20] reported lower values of CP for para and napier grass than present findings. As per the findings of Mutimura *et al*. [21], it was observed that the CP and NDF contents of hedge lucerne were less than the present findings. The chemical compositions of non-legume green fodders as reported by Datt *et al*. [22] were in agreement with present observations. Our study revealed that though all the feeds under consideration belong to the forage category of ruminant feeds, there exists wide variability in their nutritional quality and composition. The major factors that might have affected the nutritive value of such feeds are seasonality, species specificity, site of growth, soil characteristics etc., which are well supported by studies of Arzani *et al*. [23], Mahala *et al*. [24], Subhalakshmi *et al*. [25] and Teka *et al*. [26].

**Table-1:** Chemical constituents of roughage feeds (% DM).

Feeds	DM	OM	Ash	CP	EE	NDF	ADF	ADL	HC	Cellulose
Green fodders										
Maize	14.9	90.8	9.2	9.8	1.5	65.4	35.4	4.6	30.0	28.7
Oat	12.3	90.2	9.8	13.9	2.6	50.1	26.3	2.4	23.8	21.4
Sorghum	18.0	89.9	10.1	9.9	1.6	69.0	40.2	4.6	28.8	32.6
Bajra	15.9	91.0	9.0	8.1	3.1	62.5	35.0	5.6	27.5	26.8
Cowpea	16.5	91.3	8.7	17.1	3.0	48.0	31.4	8.1	16.6	20.9
Berseem	14.7	90.9	9.1	18.2	2.9	41.1	21.2	6.5	19.9	13.7
Average	15.4	90.7	9.3	12.2	2.5	56.0	31.6	5.3	24.4	24.0
Range herbages										
Para grass	23.1	88.7	11.3	10.8	1.1	75.1	49.9	6.8	25.2	40.0
Guinea grass	20.9	87.4	12.6	8.2	1.6	76.5	48.3	7.0	28.2	36.1
Hedge lucerne	23.9	90.7	9.3	19.1	1.9	49.7	37.7	10.2	12.0	24.3
Setaria grass	15.2	83.9	16.1	8.5	1.8	65.6	40.6	5.7	25.0	31.5
Hybrid napier	25.5	88.8	11.2	10.9	1.5	76.3	47.1	4.3	29.2	37.7
Average	21.7	87.9	12.1	11.5	1.6	68.6	44.7	6.8	23.9	33.9

DM=Dry matter, OM=Organic matter, CP=Crude protein, EE=Ether extract, NDF=Neutral detergent fiber, ADF: Acid detergent fiber, ADL=Acid detergent lignin, HC=Hemicellulose

### Primary CHO fractions of feeds

Total CHO content (Table-2) of all feeds was almost similar except legume feeds, which recorded lower CHO content because they had relatively higher CP content than other feeds. The NSC fraction, which represents the more digestible fraction of total CHO was higher in legume forages (cowpea, berseem and hedge lucerne). Among non-legume forages, higher NSC content was reported in oat and bajra. Forages with lower cell wall contents (NDF, ADF, cellulose, and HC) recorded higher NSC content and forages with higher cell wall contents had higher SC content. Values for total CHO, NSC and SC of the legume forages are in constitent with that reported by Chaurasia *et al*. [27]. The quantity of NSC present as starch (starch as % NSC) was higher in range herbages, while starch as % DM was higher in green fodders. ADL (% NDF) content among green fodders and range herbages were comparable. Starch (% NSC) and ADL (% NDF) content of forages varied significantly among various published reports, which was most probably due to differences in chemical constituents and the starch assay of feeds. However findings of Prusty [28] regarding primary CHO fractions of forage feeds were in agreement with this study, but findings of Das *et al*. [29] were pretty different.

**Table-2:** Primary CHO fractions of roughage feeds.

Feeds	CHO (% DM)	NSC (% DM)	SC (% DM)	NSC (% CHO)	SC (% CHO)	Starch (% DM)	Starch (% NSC)	ADL (% NDF)
Green fodders								
Maize	79.4	17.2	62.2	21.7	78.3	11.0	64.0	7.0
Oat	73.7	26.6	47.1	36.0	64.0	14.4	54.3	4.8
Sorghum	78.5	13.0	65.5	16.5	83.5	6.3	48.6	6.6
Bajra	79.8	20.0	59.8	25.1	74.9	7.4	36.9	8.9
Cowpea	71.2	31.5	39.7	44.3	55.7	11.2	35.5	16.9
Berseem	69.8	33.8	36.0	48.4	51.6	9.6	28.5	15.8
Average	75.4	23.7	51.7	32.0	68.0	10.0	44.6	10.0
Range herbages								
Para grass	76.7	8.1	68.6	10.5	89.5	7.5	93.3	9.0
Guinea grass	77.6	5.3	72.3	6.8	93.2	4.5	84.3	9.1
Hedge lucerne	69.7	29.6	40.1	42.5	57.5	11.7	39.6	20.5
Setaria grass	73.5	10.4	63.1	14.2	85.8	7.7	74.2	8.7
Hybrid napier	76.4	8.0	68.4	10.4	89.6	5.1	64.4	5.6
Average	74.8	12.3	62.5	16.9	83.1	7.3	71.2	10.6

CHO=Total carbohydrate, NSC=Nonstructural carbohydrate, SC: Structural carbohydrate, DM=Dry matter, ADL=Acid detergent lignin, NDF=Neutral detergent fiber

### Primary protein fractions of feeds

Primary protein fractions (NDICP, ADICP, NPN and SP) of forage feeds are presented in Table-3. NDICP (% DM and % CP) content of range herbages was higher than green fodders while ADICP (% DM and % CP) content was comparable between two groups of forages. ADICP content represents that fraction of feed protein, which is neither available to microbes nor to the animal in case of ruminants. Low ADICP (% CP) content was recorded in oat and berseem than other feeds. Lower values of NPN content was reported in bajra and setaria grass. Oat, berseem and hedge lucerne contained higher SP values indicating their superior protein availability in the rumen. SP (% CP) content in green fodders was higher than range herbages while NPN (% SP) content in range herbages was higher. Forages like bajra, oat and berseem had lower NPN (% SP) contents, which suggested that these feeds had higher soluble TP content. Reports of Kamble *et al*. [6] and Gupta *et al*. [30] regarding primary protein fractions were not in agreement with our results. This was probably because of the differences in chemical composition of feeds and estimation procedures.

**Table-3:** Primary protein fractions of roughage feeds (% DM).

Feeds	NDICP		ADICP		TP	NPN	SP	IP	SP (% CP)	NPN (% SP)
	
	% DM	% CP	% DM	% CP						
Green fodders										
Maize	3.2	32.6	1.5	15.3	7.5	2.3	2.8	7.0	28.6	82.1
Oat	2.9	20.9	0.8	5.7	11.8	2.1	6.4	7.5	46.0	32.8
Sorghum	3.5	35.3	1.5	15.1	8.0	1.9	4.3	5.6	43.4	44.2
Bajra	2.7	33.3	1.4	17.3	7.3	0.8	2.7	5.4	33.3	29.6
Cowpea	8.4	49.1	3.0	17.5	15.3	1.8	2.9	14.2	16.9	62.1
Berseem	5.1	28.0	1.6	8.8	16.4	1.8	5.4	12.8	29.7	33.3
Average	4.3	33.2	1.6	13.3	11.1	1.8	4.1	8.8	33.0	47.4
Range herbages										
Para grass	6.4	59.3	1.8	16.7	7.3	3.5	3.9	6.9	36.1	89.7
Guinea grass	4.1	50.0	1.8	21.9	6.5	1.7	2.4	5.8	29.3	70.8
Hedge lucerne	9.6	50.3	2.3	12.1	16.9	2.2	5.3	13.8	27.7	41.5
Setaria grass	2.5	29.4	1.1	12.9	8.0	0.5	1.1	7.4	12.9	45.5
Hybrid napier	6.7	61.5	2.0	18.3	7.6	3.3	3.9	7.0	35.8	84.6
Average	5.8	50.1	1.8	16.4	9.3	2.2	3.3	8.2	28.4	66.4

NDICP=Neutral detergent insoluble CP, ADICP=Acid detergent insoluble CP, TP=True protein, NPN=Non protein nitrogen, SP=Soluble protein, IP=Insoluble protein, DM=Dry matter, CP=Crude protein

### CNCPS CHO and protein fractions

When CNCPS CHO fractions of forage feeds (Table-4) were interpreted, it was observed that legume forages contained higher CA fraction indicating that these feeds were better sources of fermentable CHO to ruminants. This findings regarding legume forages were in agreement with findings of Kamble *et al*. [6] and Gupta *et al*. [30]. Para and setaria grass contained lower CA fraction. Amount of fraction CB_1_ was comparable between green fodders and range herbages. Sorghum and bajra among green fodders had lower CB_1_ content while among range herbages para and guinea grass had lower CB_1_ content. In typical ruminant diet, the amount of fraction CB_2_ is very important as this fraction represents the available cell wall portion of ruminant feeds. Obviously legume forages contained lower CB_2_ fraction as these feeds are high in protein content and low in NDF content. Among other feeds, range herbages contained higher fraction CB_2_ than green fodders reflecting their high cell wall availability to ruminants. Fraction CC is the lignin bound cell wall content of a feed. Hence this fraction is indigestible both by ruminal microbes and the animal itself. Feeds with low CC fraction will be of superior quality in terms of CHO supply to ruminants and vice-versa. On this aspect, forages like oat and hybrid napier were found to be better feeds. Findings of Trivedi *et al*. [5] regarding various CHO fractions of forages though were different from our results, but they were highly comparable. On analyzing the CNCPS protein fractions of test feeds (Table-5), it was found that forages like bajra, berseem, setaria grass and hedge lucerne contained lower PA fraction, which was direct reflection of their low NPN (% SP) values. The PC fraction is that fraction of feed CP which cannot be degraded by both ruminal microbes and the animal itself, thus possesses practically no feeding protein value [31]. Average PC content of range herbages were slightly more than that of green fodders. Feeds like oat and berseem had lower PC contents reflecting their high protein bioavailability. The fractions PB_2_ and PB_3_ are degraded in the rumen to a lesser extent than PA and PB_1_, thus feeds with high PB_2_ and PB_3_ content will have more by-pass protein value. All legume forages had higher PB_2_ + PB_3_ content while among non-legume feeds maize fodder, and the setaria grass contained higher PB_2_ + PB_3_ content. Very similar observations were made by Kamble *et al*. [6] and Gupta *et al*. [30].

**Table-4:** CNCPS carbohydrate fractions of roughage feeds (% CHO).

Feeds	CA	CB1	CB2	CC
Green fodders				
Maize	7.8^d^	13.9^c^	64.5^a^	13.8^d^
Oat	16.5^c^	19.5^a^	56.1^b^	7.9^e^
Sorghum	8.5^d^	8.0^d^	69.4^a^	14.1^d^
Bajra	15.8^c^	9.3^d^	58.2^b^	16.7^c^
Cowpea	28.6^b^	15.7^b^	28.3^c^	27.4^a^
Berseem	34.5^a^	13.7^c^	29.5^c^	22.3^b^
Average	18.6	13.4	51.0	17.0
Range herbages				
Para grass	0.7^d^	9.8^c^	68.2^ab^	21.3^bc^
Guinea grass	1.1^d^	5.7^d^	71.6^a^	21.6^b^
Hedge lucerne	25.7^a^	16.8^b^	22.4^d^	35.1^a^
Setaria grass	3.7^c^	10.5^c^	67.3^b^	18.5^c^
Hybrid napier	10.6^b^	19.2^a^	59.3^c^	10.9^d^
Average	8.3	12.4	57.8	21.5

Means bearing different superscripts in the same column differ significantly. (*p<0.05), CNCPS=Cornell Net Carbohydrate and Protein System, CHO=Total carbohydrate

**Table-5:** CNCPS protein fractions of roughage feeds (% CP)

Feeds	PA	PB1	PB2	PB3	PC
Green fodders					
Maize	23.5^a^	5.1^d^	38.8^b^	17.3^c^	15.3^b^
Oat	15.1^c^	30.9^a^	33.1^c^	15.2^c^	5.7^d^
Sorghum	19.2^b^	24.2^b^	21.3^d^	20.2^b^	15.1^b^
Bajra	9.9^d^	23.4^b^	33.4^c^	16.0^c^	17.3^a^
Cowpea	10.5^d^	6.4^d^	34.0^c^	31.6^a^	17.5^a^
Berseem	9.9^d^	19.8^c^	42.3^a^	19.2^bc^	8.8^c^
Average	14.7	18.3	33.8	19.9	13.3
Range herbages					
Para grass	32.4^a^	3.7^c^	4.6^c^	42.6^a^	16.7^b^
Guinea grass	20.7^b^	8.6^b^	20.7^b^	28.1^c^	21.9^a^
Hedge lucerne	11.5^c^	16.2^a^	22.0^b^	38.2^b^	12.1^c^
Setaria grass	5.9^d^	7.0^b^	57.7^a^	16.5^d^	12.9^c^
Hybrid napier	30.3^a^	5.5^c^	4.6^c^	43.2^a^	16.4^b^
Average	20.2	8.2	21.9	33.7	16.0

Means bearing different superscripts in the same column differ significantly (*p<0.05), CNCPS=Cornell Net Carbohydrate and Protein System, CP=Crude protein

## Conclusion

Based on the findings of the above study it was concluded that among typical ruminant forage feeds, oat fodder and hybrid napier grass were better feeds of ruminants from CHO supply point of view while oat and berseem fodders were found to be good forage protein sources to ruminants. Maize fodder was evaluated to be a good feed with more by pass protein value. All the above feeds are extensively used in our country as forages for ruminant feeding and their preferential selection as animal forage sources are established by CNCP system. Therefore, this CNCPS model could be successfully implemented for nutritive evaluation of forage feeds of Indian origin in terms of their CHO and protein fractions though sufficient database should be developed before its applicability in dairy ration formulation.

## Authors’ Contributions

SSK and CD designed the plan of the present study. LKD and DK carried out the experimental work. Manuscript preparation along with data analysis was done by LKD. All authors read and approved the final manuscript.
